# The Role of RBC Oxidative Stress in Sickle Cell Disease: From the Molecular Basis to Pathologic Implications

**DOI:** 10.3390/antiox10101608

**Published:** 2021-10-13

**Authors:** Qinhong Wang, Rahima Zennadi

**Affiliations:** Duke Comprehensive Sickle Cell Center and Division of Hematology, Department of Medicine, School of Medicine, Duke University, Durham, NC 27710, USA; qinhong.wang@duke.edu

**Keywords:** reactive oxygen species, NADPH oxidase, red blood cells, sickle cell disease

## Abstract

Sickle cell disease (SCD) is an inherited monogenic disorder and the most common severe hemoglobinopathy in the world. SCD is characterized by a point mutation in the β-globin gene, which results in hemoglobin (Hb) S production, leading to a variety of mechanistic and phenotypic changes within the sickle red blood cell (RBC). In SCD, the sickle RBCs are the root cause of the disease and they are a primary source of oxidative stress since sickle RBC redox state is compromised due to an imbalance between prooxidants and antioxidants. This imbalance in redox state is a result of a continuous production of reactive oxygen species (ROS) within the sickle RBC caused by the constant endogenous Hb autoxidation and NADPH oxidase activation, as well as by a deficiency in the antioxidant defense system. Accumulation of non-neutralized ROS within the sickle RBCs affects RBC membrane structure and function, leading to membrane integrity deficiency, low deformability, phosphatidylserine exposure, and release of micro-vesicles. These oxidative stress-associated RBC phenotypic modifications consequently evoke a myriad of physiological changes involved in multi-system manifestations. Thus, RBC oxidative stress in SCD can ultimately instigate major processes involved in organ damage. The critical role of the sickle RBC ROS production and its regulation in SCD pathophysiology are discussed here.

## 1. Introduction

Sickle cell disease (SCD) is a hereditary autosomal recessive red blood cell (RBC) disorder resulting from a point mutation in the β-globin gene, resulting in the production of the sickle hemoglobin (Hb S) due to substitution of valine for glutamic acid at the sixth amino acid position [1,2]. SCD affects approximately 100,000 people in the United States and millions worldwide, especially those of African ethnicity [3,4]. In SCD, Hb S tends to polymerize under conditions of low oxygen saturation. RBCs containing the Hb S polymers are inclined to convert the normal biconcave disc shape of RBCs into a rigid, irregular sickled shape which could block blood vessels in microcirculation, thus impairing the delivery of oxygen to tissues. The repeated polymerization of Hb S leads to a cyclic cascade inciting sickle RBC and other blood cell adhesion-promoting episodic vaso-occlusive events known as “pain crises” with subsequent ischemia-reperfusion injury [5,6,7], intravascular hemolysis, multiple organ damage, and short lifespan [8].

Reactive oxygen species (ROS) are produced because of intracellular catabolism that requires oxygen as a terminal electron acceptor. Under normal conditions, there is a balance between oxidant and antioxidant systems, preventing oxidative damage [9,10]. Oxidative stress results from the imbalance between oxidant and antioxidant systems, which triggers a cascade of reactions damaging membrane lipids, proteins, and DNA, causing a series of pathobiological events [11,12]. One of the most prominent phenomenon of sickle RBCs is that the RBCs contain high levels of ROS and oxidative stress due to both the autoxidation of Hb S and NADPH oxidase (NOX)-mediated ROS production [13,14,15]. A body of emerging evidence indicates that ROS within the sickle RBC play an essential and contributing role in the SCD pathophysiology. In this review, we will focus on how ROS produced by NOX enzymes, in contribution with Hb S autoxidation within sickle RBCs, affect interactions of these cells, with the vascular endothelium and other blood cells promoting vaso-occlusion, activation of coagulation, hemolysis-related anemia, activation of the complement system, endothelial dysfunction and tissue injury, and further inflammation, all of which promote SCD vascular pathology.

## 2. ROS Generation in Sickle Red Blood Cells

**Figure 1:**
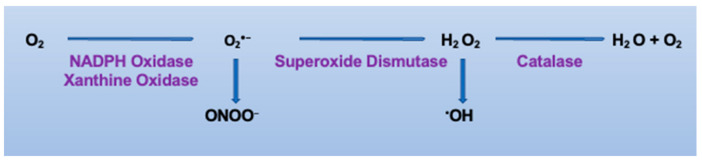
Generation of reactive oxygen species. Oxygen readily accepts free electrons produced by normal oxidative metabolism, generating the unstable superoxide anions (O_2_^•−^), which are scavenged by superoxide dismutase (SOD) and transformed into hydrogen peroxide (H_2_O_2_) or rapidly converted into peroxynitrite (ONOO^−^) when reacting with bioactive nitric oxide (^•^NO). The H_2_O_2_ converts to water by catalase. In the Harber–Weiss reaction, hydroxyl radicals (^•^OH) are generated in the presence of H_2_O_2_ and iron ions.

ROS include free radical and non-free radical oxygen intermediates such as superoxide (O_2_^•−^), the hydroxyl radical (^•^OH), and hydrogen peroxide (H_2_O_2_) (Figure 1). Production of O_2_^•−^ and H_2_O_2_ can lead to the formation of the highly cytotoxic ^•^OH in the presence of ferrous ion. Furthermore, O_2_^•−^ has the ability to react rapidly with nitric oxide (^•^NO) to form peroxynitrite (ONOO^−^), a very strong nitrating and oxidizing compound [16]. These highly reactive ^•^OH and ONOO^−^ radicals can initiate membrane protein and lipid oxidation to generate more complex radicals. ROS are generated in a variety of mammalian cells in the context of both health and disease [17]. Under physiological conditions, ROS are critical mediators of the cell signaling involved in cellular and biologic functions, such as cell proliferation, migration, wound healing, and angiogenesis. In pathological settings, however, ROS are generated in excess and play a negative role by inducing cellular/tissue damage. Thus, redox homeostasis maintenance is essential for cell survival [9,10].

RBCs are highly sensitive towards ROS that oxidatively damage membrane macromolecules, ultimately compromising oxygen delivery and leading to both aging and cell death [9]. The antioxidant system in RBCs consisting of enzymatic antioxidants, including catalase (Cat), superoxide dismutase (SOD), glutathione peroxidase (GPx), glutathione reductase (GR), and peroxiredoxin-2 (Prx-2) [10,18,19,20], and non-enzymatic low molecular-weight antioxidants (reduced and oxidized) either produced intracellularly (glutathione (GSH)/glutathione disulfide (GSSG) and NADH/NADPH) or up-taken by the cells (α-tocopherol (Vitamin E), ascorbate (ASC), bioflavonoids, and selenium) [21] can neutralize ROS overload, thus reducing oxidative stress.

In SCD, however, multiple mechanisms have been suggested to be implicated in an elevated oxidative burden in sickle cell patients. Sickle RBCs are the primary source of oxidative stress in SCD and the source of sickle RBC ROS is intricate. The intracellular ROS exert a tremendous impact on the physiological functions of the cell. In sickle RBCs, the autoxidation of the Hb S is primarily responsible for the excessive ROS generation due to Hb S high instability [9,13]. Although oxygenated hemoglobin is referred to as a relatively stable molecule, it can still physiologically be autoxidized to methemoglobin [22]. Hb S has the ability to undergo autoxidation in the presence of oxygen to produce ROS within sickle RBCs [13,23] at a rate higher than that produced by normal Hb (Hb A) [13,24]. It is also well-known that the vicious cycles of sickling and unsickling result in high amounts of ROS production [25]. ROS located at the plasma membrane are not readily accessible to the cytoplasmic antioxidant system [9] and subsequently easily oxidize membrane lipids and proteins, causing extensive oxidative damage [26]. Apart from Hb S oxidation, ROS can also be generated enzymatically by NOX enzymes [14,15,27], which are regulated by PKC, RacGT, and Ca^2+^ signaling in sickle RBCs [14]. NOX-derived ROS that accumulate within sickle RBCs mediate sickle cell adhesion to the vascular endothelium and vaso-occlusion [15,27], and further contribute to the oxidative damage to the subcellular structures, leading to increased RBC fragility and hemolysis [28]. Abnormal mitochondrial retention in sickle RBCs also contributes to excessive levels of ROS in these cells [29]. In addition, lower levels of the antioxidant enzymes SOD and Cat were detected in sickle RBCs compared to normal RBCs [30]. It was also reported that the glutathione system is significantly impaired, indicated by reduced GSH_total_, GSH_reduced_, and GSSG values in sickle RBCs compared to the normal controls [31]. Consequently, this leads to an imbalance between prooxidants and both the enzymatic and non-enzymatic antioxidant defense system, which compromises the redox homeostasis [32,33,34,35]. Indeed, a continuous enhanced Hb autoxidation in combination with NOX activation causes ROS accumulation, which rapidly overwhelms the available membrane and non-membrane antioxidants such as GPx, Prx-2, thioredoxin, and GSH [10,19,31,36,37,38,39]. Peroxiredoxin-2 is essential for preventing hemolytic anemia from oxidative stress through maintaining hemoglobin stability, while GSH and thioredoxin are important antioxidant molecules that maintain the balance of oxidation and antioxidation processes in sickle RBCs [40,41]. Importantly, the fact that these enucleated cells are deprived of protein synthesis machinery leaves these cells even more susceptible to oxidative stress-mediated damage [42]. Thus, in SCD, the antioxidant system in sickle RBCs has a limited ability to neutralize the endogenous ROS in excess. The un-neutralized ROS and the ensuing oxidative stress in the sickle RBCs by damaging membrane structures can mainly impact cellular functional activities [43]. Disruption of the organizational cell structure during oxidative stress typifies an ultimate mechanism involved in system failure [21]. Damaged and apoptotic RBCs are normally phagocytosed by macrophages and cleared from the circulation to maintain RBC homeostasis [44,45]. However, in SCD, phagocytosis is ineffective [46], favoring accumulation in the circulation of apoptotic RBCs and likely hemolysis-free Hb, heme-loaded vesicles, and heme. Thus, oxidative damage to the sickle RBCs impairs hemorheological properties [47,48]. This leads to sickle RBCs up-taking, in addition to ROS being released from neutrophils [49], macrophages, and endothelial cells [50,51,52], causing further damage to the cells.

## 3. Endogenous Oxidative Stress and Sickle Red Blood Cell Structures

Oxidative stress is a key modulator of RBC rheological properties. Oxidative stress affects RBC membrane (i.e., lipid peroxidation and protein oxidation) and caspase 3 activation [53]. In SCD, oxidative stress burden is high in sickle RBCs [54,55], with a further rise during painful vaso-occlusive crises [56]. Hb-dependent oxidative reactions cause posttranslational modifications of β-globin with subsequent oxidation of Hb βCys93 and ubiquitination of both Hb βLys96 and βLys145, resulting in RBC membrane changes of band 3 and the ubiquitination of proteins [57,58]. Ubiquitination of Hb and phosphorylation of band 3, a prerequisite step for band 3 clustering and microparticle (MP) release, were shown to be more abundant in samples of SCD patients [58]. The ROS generated due to Hb autoxidation also reacts with membrane lipids and proteins due to their ideal location, causing oxidation of lipids and membrane proteins, consequently altering conformation of cytoskeleton proteins and intensifying membrane fragility [9,59,60,61,62]. Several studies have evidenced increased lipid peroxidation levels in sickle erythrocytes and other tissues due to oxidative stress [37,63,64,65]. Accumulation of lipid peroxidation in RBCs affects band 3-associated enzymes such as phosphofructokinase and glyceraldehydes-3-phosphate dehydrogenase [64]. Oxidative reaction-mediated activation of caspase 3 in RBCs can also partially degrade band 3 [66,67], affecting band 3 interactions with cytosolic proteins as well as the linkage to ankyrin and the cytoskeleton. As a result, phosphatidylserine (PS), a negatively charged phospholipid normally present on the cytoplasmic side, is exposed on the surface of the RBC membrane [68]. This dramatic rearrangement of the membrane is involved in a concomitant decrease in membrane deformability. Hemichromes, the intermediate products of Hb oxidative denaturation, also have a high affinity for the cytoplasmic domain of band 3, causing the oxidative cross-linking through disulphide bonds [69]. Band 3 oxidation generates oxidative stress, fostering phosphorylation of the cytoplasmic domain of band 3 by a sequential action of tyrosine kinase Syk and tyrosine kinase(s) belonging to the Src family [70]. Subsequently, tyrosine phosphorylation promotes dissociation of band 3 from the spectrin-actin skeleton [69,71]. Tyrosine phosphorylation of the multifunctional transmembrane protein band 3 has been implicated in several erythrocyte functions and disorders. In SCD, this leads to membrane blebbing and the production of MPs [72]. Additionally, a higher erythrocyte aggregation tendency and increased acetyl cholinesterase (AChE) activity is evidenced when band 3 is phosphorylated, but not when it is dephosphorylated [73,74]. Oxidative stress in sickle RBCs affects not only the membrane but impairs cytoskeletal proteins as well, including spectrin, actin, and protein 4.1 [75]. Spectrin oxidation disrupts its binding to actin or to proteins that link the membrane and cytoskeleton, such as protein 4.1 [76]. This compromises the membrane stability in the interaction between the membrane and cytoskeleton, and thereby increases membrane susceptibility to hemolysis. Additionally, our studies have shown increased phosphorylation by ERK1/2 of cytoskeletal proteins, α and β-adducins and dematin at the ERK1/2 consensus motif, and protein 4.1, promoting sickle RBC adhesion to the endothelium [15,77]. Increased phosphorylation of adducin at Ser-726, a target of PKC, has been evidenced in sickle RBCs [78]. The phosphorylation of adducin promotes dissociation of spectrin from F-actin, thus disrupting membrane integrity. PKC also regulates the activity of NOX-dependent ROS generation in sickle RBCs [14,15]. In the same manner, disruption of the physiological asymmetry of phospholipids causes PS exposure to the outer membrane [79,80]. Under physiological conditions, RBC exposing PS are recognized and removed by scavenging receptors in macrophages, which engulf and degrade the PS-exposing cells [42]. Yet, PS-positive RBCs have been observed in SCD [81]. The repeated cycles of sickling and unsickling is another factor that leads to abnormal PS exposure on sickle RBCs. Cycles of sickling/unsickling disturb the membrane phospholipid asymmetry, also causing micro-vesicle formation [82]. Furthermore, oxidative stress inhibits the activity of Ca-ATPase, which is involved in regulating calcium (Ca^2+^) levels [42,83]. Enhanced intracellular Ca^2+^ in RBCs activates the Gardos channel and outflow of potassium from the cells, impairing cation homeostasis, which induces shrinkage of the cell and lessens deformability [84,85]. In addition, accumulation of Ca^2+^ within sickle RBCs can trigger RBC membrane-scrambling, resulting in PS exposure and possibly in membrane-bubbling and release of MPs [86]. It has also been described that reduced GSH, a thiol-containing antioxidant agent, affects free radicals scavenging and both membrane protein and lipid protection from free radical-mediated oxidation [31,65,87]. Sickle RBC GSH decline is due to the reduced expression of the proteins involved in GSH synthesis and its reduction [88,89]. Previously, erythrocyte AChE activity, which can be altered by oxidative stress [90,91], was reported as a biomarker of membrane integrity [92]. In SCD, both AChE and ATPase activities were markedly higher in the erythrocyte membrane from sickle cell patients homozygous for Hb S (SS) than in those from individuals with sickle cell traits (AS) or normal (AA) controls [93]. These higher values of AChE and ATPase activities in sickle RBCs may be a consequence of the abnormally high cation levels in these sickle cells [93]. Large amounts of AChE are similarly present in erythrocyte membrane exovesicles [94,95]. Moreover, SCD is associated with a defective autophagy process [96]. Unsuccessful removal of PS-decorated vesicles causes the elevation in PS-exposed RBCs in SCD. High numbers of circulating PS-positive RBCs has been described after splenectomy and in patients with hemoglobinopathies [80]. Loss of autophagy in erythroid cells leads to a defect in mitochondria removal and severe anemia in vivo [97,98]. The autophagy process, when impaired, contributes to the increased oxidative stress and accumulation of damaged RBCs, cell organelles, heme-loaded vesicles, and oxidative stress-mediated protein aggregates in the circulation, possibly contributing to the development of several disease-dependent symptoms [99,100,101]. Indeed, in SCD, oxidative stress in RBCs instigates a slow buildup of damaged products, such as oxidized proteins, as well as advanced glycation and peroxidation end products. Thus, sickle RBC-dependent vulnerability towards oxidative stress and increased levels of oxidative stress biomarkers in these cells affect membrane structure and function, with the loss of membrane properties, decreased deformability, cell senescence, and hemolysis. Consequently, a variety of pathological events follow, including vaso-occlusion, generation of RBC-derived MPs, chronic hemolysis, hypercoagulation, vascular endothelial cell dysfunction, ischemia-reperfusion organ injury, and inflammation [8,11,27,28,29]. In addition, oxidative stress-dependent hemolysis-associated free Hb is also one of the vital factors contributing to autonomous and non-autonomous injury to the endothelium [102] as well as endothelial cell death [103].

## 4. The Role of Sickle RBC ROS in the Adhesion to Endothelium and Vaso-Occlusion

**Figure 2:**
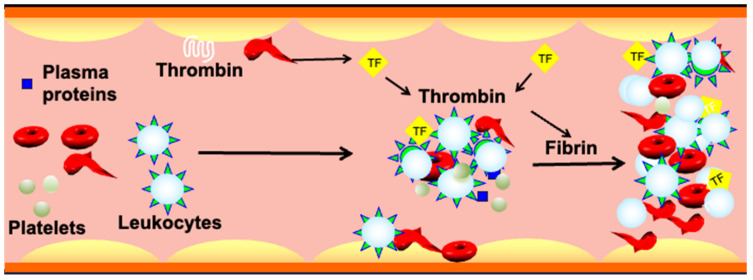
The role of sickle RBCs in vaso-occlusion. Sickle RBCs interact with and activate the vascular endothelium, leukocytes, and platelets, and bind to coagulation factors and plasma proteins forming aggregates. These adhesive interactions progressively induce the occlusion of blood vessels and blood stasis.

In SCD, recurrent vaso-occlusive episodes (VOCs), the hallmark of the disease, contribute to morbidity and premature mortality, primarily due to acute systemic painful vaso-occlusive crises, “pain crises” [104,105,106], and progressive irreversible ischemic end-organ damage. The pathophysiology of vaso-occlusion is complex and multifactorial, involving deoxygenation-dependent Hb S polymerization and sickling [6,107,108,109]; altered cell adhesion [7,79,110,111,112,113,114]; and activation of leukocytes [113,115], the endothelium [116,117,118,119,120] and the coagulation cascade [121,122,123], oxidative stress [11,49,55,124,125,126], and both intracellular and extracellular signaling pathways (Figure 2) [15,77,127,128,129,130,131]. Although all these factors act in a positive feedback-loop to drive the pathophysiology of SCD, the gravity and impact of each of these factors in initiating VOCs appear to vary.

Abnormal adhesion of sickle RBCs to the endothelium has been postulated to initiate vaso-occlusion, which occurs primarily in the microcirculation [132,133,134]. In fact, Hebbel and colleagues have shown that sickle RBCs adhere to cultured endothelial cells [135] and the degree of sickle RBC adhesion correlates with clinical disease severity [133]. Since these initial studies, many groups have studied the mechanisms of sickle RBC interactions with other cell types. Sickle RBCs expose multiple adhesion molecules involved in the adhesion of these sickle cells to the extracellular matrix (ECM), endothelial cells, leukocytes, and other cellular ligands [113,128,129,130,131,136,137,138,139]. Sickle RBCs, by interacting with leukocytes, activate leukocyte adhesion to the endothelium [113]. Activated leukocyte interactions with endothelial cells contribute to sickle RBC recruitment and adhesion to stationary leukocytes, further occluding small vessels [114]. Sickle RBCs can also form multi-cellular aggregates with other blood cells, promoting vaso-occlusive events [140,141,142]. In contrast, although normal RBCs express all the adhesion receptors expressed by mature sickle RBCs, they exhibit few adhesive phenotypes [128,137,139,143,144,145].

Oxidative stress is prominent in SCD [11,13] and is increasingly accepted as a component of vaso-occlusive episodes [146,147,148]. Lately, the considerable complexities of oxidative mechanisms in sickle RBCs have been shown to also involve an intra-erythrocytic positive feedback loop created by NOX enzymes’ dependent-ROS production, the mitogen-activated protein kinases (MAPKs) known as the extracellular signal-regulated kinases ERK1/2 and G protein-coupled receptor kinase 2 (GRK2), to mediate the adhesion of these sickle cells to the vascular endothelium both in vitro and in vivo [15]. This sickle RBC signaling-mediated adhesive phenotype can be up-regulated by hypoxia/reoxygenation (H/R), implicating the activation of the RBC adhesion molecules LW (ICAM-4 and CD242) blood group glycoprotein and CD44 [15], and is disrupted by manganese (Mn) porphyrins [27]. Plasma from patients with SCD and isolated cytokines, such as transforming growth factor β1 and endothelin 1, also enhance sickle RBC NOX activity and increase ROS generation [14]. Nonetheless, ROS generation, due to iron decompartmentalization and Hb S autoxidation in particular [149], may also provide an additional activating factor of ERK1/2 and GRK2 within the sickle RBC. Previous studies have postulated that deoxygenation-induced sickling destabilizes membrane phospholipid asymmetry to enhance PS availability on the surface of sickle RBC membranes [81]. Sickle RBC PS exposure plays an important role in the adhesion to the endothelium [79]. Thus, it is now clear that enhanced ROS generation in sickle RBCs via at least endogenous NOX, activated by exposing the cells to H/R, mediates adhesive interactions with the vascular endothelium and with vaso-occlusion in SCD.

The substantial oxidative stress in sickle RBCs further increases Hb S oxidation, which could contribute to cell membrane damage and premature hemolysis [150]. Sickle RBCs are susceptible to exogenous free radicals generated from hemolysis-free Hb, heme, a hydrophobic, iron-containing molecules, and iron, which can also trigger activation of the intracellular sickle RBC feedback loop through activation of ERK1/2 and GRK2, leading to anomalous sickle RBC adhesion and episodic vaso-occlusive crises. It may be anticipated that abnormal rheologic properties would participate in these frequent vaso-occlusive crises as well. Furthermore, extracellular Hb, heme, heme-free iron, and microparticles in plasma [151] trigger severe oxidative stress in blood cells and vessels, leading to vascular dysfunction and vaso-occlusion in SCD [52,152,153]. Increased ROS in the bloodstream signals to activate endothelial nuclear factor κB (NF-кB), thereby eliciting the expression of the adhesion molecule (e.g., VCAM-1, ICAM-1, and P-selectin, the latter of which can be directly up-regulated by ROS) [146,152], promoting leukocyte recruitment, sickle RBC/leukocyte interactions, transient vaso-occlusion, and H/R injury. Additionally, free heme-associated oxidative stress elicits proinflammatory and prothrombotic phenotypes [154,155,156,157,158]. Furthermore, extracellular Hb scavenges ^•^NO, consequently decreasing NO bioavailability [159], which results in vascular dysfunction and vaso-restriction. It has further been shown that MPs bud from sickle RBCs after sickling concurrent with the unsickling phase [158,160,161]. They contain Hb like that of the parent RBCs [162]. More importantly, sickle RBC-derived MPs possibly display the exaggerated oxidant generation that is present in sickle RBCs [14,15,23]. These sickle RBC-derived MPs widely distribute their oxidatively active heme iron [163] to the vascular endothelium [162], which substantially increases oxidative stress-associated vascular dysfunction and activation of complement-mediated vascular damage, contributing to the overall vaso-occlusive crisis [161,162]. MPs are present at levels three to ten-folds higher in SCD patients during steady state than in health control subjects and those levels can further increase by up to three-folds during VOCs [72,164]. Thus, the recurrent sickle RBC adhesive events caused by increased ROS production and oxidative stress, together with hemolysis and progressive endothelial damage, create an extracellular positive vicious loop that guides the profound manifestations and serious outcomes in SCD.

## 5. The Contribution of Sickle RBC ROS to Hemolysis

The sickle RBCs can often break when it passes through the blood vessels, causing premature destruction of the cells. Sickle RBCs remain in blood circulation for approximately 20 days versus 120 days for normal RBCs. Severe hemolysis in SCD leads to an increase in the proportion of reticulocytes [165,166,167]. About one-third of sickle RBC destruction occurs within the vessels, generating high levels of circulating hemoglobin and iron [104,168,169]. The mechanisms of oxidative stress-mediated hemolysis of sickle RBCs are not clear. One possibility is that the intra and extra- erythrocytic ROS cause accelerated intravascular hemolysis through induction of lipid peroxidation, cell membrane damage, and fragility [13,87]. Another possibility is that PS-exposing erythrocytes are hydrolyzed by phospholipase A2, resulting in increased intravascular hemolysis. These sickle cells could also be recognized by macrophages with PS-specific receptors breaking erythrocytes [170,171]. Sickle RBCs have a high level of ROS-related PS exposure, suggesting that oxidative stress might play an important role in intravascular hemolysis [80,87]. In SCD, RBC-derived MPs may be, in part, products of hemolysis as well [172]. Sickle RBC-derived MPs correlate with the reticulocyte count, plasma Hb, and lactate dehydrogenase, and inversely with fetal hemoglobin level [72,158,173].

The progressive release of redox-active iron and heme during hemolysis, and RBC-derived MPs into the bloodstream, stimulates a chain reaction that is toxic to the vasculature, contributing to the development of vascular disease [174]. Hemolysis-free heme, a highly inflammatory agent, activates the innate immune pattern recognition receptor, toll-like receptor (TLR) 4 on monocytes/macrophages, and endothelial cells [52,175]. Heme activation of TLR4 on lung endothelial cells promotes acute chest syndrome in the transgenic SCD mice [176] as well as NETosis, comprised of decondensed chromatin and DNA from activated neutrophils, known as neutrophil extracellular trap (NET) formation [177]. Heme derived from sickle RBCs can activate TLR4 independently of LPS, leading to oxidant production, inflammation, and vaso-occlusion [52]. Heme promotes vaso-occlusion via rapid mobilization of the Weibel–Palade body (WPB), P-selectin, and von Willebrand factor (VWF) onto endothelial cell and vessel wall surfaces, alongside promoting activation of NF-κB [52]. In SCD, depletion in the plasma high-affinity heme-binding protein hemopexin favors heme-mediated TLR4 activation [52,178]. Hemolysis is also accompanied by the release of a number of molecules with inflammatory potential, called damage-associated molecular patterns (DAMPs), such as adenosine 5′ triphosphate (ATP) [179]. In addition to acting as universal energy source, ATP acts in a paracrine fashion to increase the vascular caliber, resulting in increased oxygen delivery. Defects in ATP’s vasoactive properties could contribute to the perfusion abnormalities in skeletal muscle [180]. Extracellular ATP can be rapidly converted to adenosine by ectonucleotidases. The role of adenosine-signaling during sickle cell disease is complicated. Although studies have indicated a protective role of adenosine purine molecule-stimulated Adora2a-signaling via attenuating ischemia, reperfusion injury–associated pulmonary inflammation, and T cell activation [181], other studies suggest that the stimulation of the Adora2a adenosine receptor plays a functional role by promoting erythrocyte sickling [182].

## 6. The Contribution of ROS in Sickle RBCs to Inflammation and Vascular Damage

Oxidative stress is generally believed to make a significant contribution to ischemic diseases and postischemic reperfusion injury. In SCD, ischemia occurs as the result of the interruption of the blood supply, causing hypoxia due to vaso-occlusion, and reperfusion injury is defined as the tissue damage following vaso-occlusion resolution and consequent reperfusion of the tissue [183]. As discussed above, sickle RBC ROS in excess mediates RBC adhesion and vaso-occlusion, and can be released by the RBCs while in contact with endothelial cells. Therefore, it is important to emphasize the fact that ROS produced by sickle RBCs play an important part in the pathological processes of inflammation caused by ischemia-reperfusion due to recurrent vaso-occlusive events [184,185,186,187], as well as is involved in ischemia-induced tissue damage and reperfusion injury. Ischemia and reperfusion are in themselves highly inflammatory mechanisms. The chronic proinflammatory state is one of the most characteristic features in SCD, even in the clinically asymptomatic phase [185]. The relationship between oxidative stress and the pro-inflammatory state in SCD is complicated, and it is most likely bidirectional. Pro-inflammatory cytokines, such as TNFα, IL-6, and IL-1, and the production of IFNγ, have been shown to be enhanced in SCD [188,189,190]. Although the produced pro-inflammatory cytokines promote the generation of additional ROS, increased recruitment and the adhesion of leukocytes, neutrophils in particular, to the endothelium [191] can exacerbate endothelial oxidative stress [49,177,192]. Recent evidence has involved endothelial NOX-derived superoxide in the adhesion of leukocytes and platelets in cerebral venules of sickle mice [51]. Increased leucocyte influx is both an important inflammatory marker and a major source of ROS due to the superoxide-producing enzymes’ NOXs [114,193,194]. Thus, NOX enzymes, the major superoxide-producing enzymes in sickle RBCs, endothelial cells, and leukocytes, are a potentially noteworthy source of ROS in SCD and they are likely critical in triggering the SCD-related inflammatory state, especially the absolute implication of soluble circulating xanthine oxidase (XO) and endothelial-associated XO in ROS generation in the vasculature is still unclear.

During ischemia, pre-apoptotic RBCs and other blood cells release damage-associated molecular patterns (DAMPs), such as ATP (hemolysis and necrotic cells), heme (hemolysis), high-mobility group box 1 (HMGB1) (necrotic cells), and heat shock proteins (hemolysis and necrotic cells), parameters of which are increased in SCD [195,196]. These DAMPs can promote multiple inflammatory pathways, including NET formation through HMGB1 and TLR4 interactions, and the assembly of inflammasomes, which are multiprotein cytoplasmic complexes involved in mediating cellular inflammation in response to various damaging agents [197,198]. ROS were demonstrated to induce the assembly and activation of inflammasomes [199,200]. In addition, prolonged ischemia (a restriction in tissue blood supply, causing a deficiency of oxygen delivery by RBCs) can lead to necrosis and cell death. Although what directs the cell to either chronic inflammation or apoptosis is so far uncertain, it is expected that the chosen outcome is under tight control [201]. During restoration of the blood flow subsequent to blood stasis due to vaso-occlusion, further tissue damage takes place as a result of reoxygenation, as reflected by the increased production of ROS and the calcium overload [183]. Indeed, if some oxygen-derived compounds, such as ROS and oxygen-derived free radicals, can participate as helpful molecules in cell-signaling processes, they can also exert deleterious effects, contributing to the pathogenesis of endothelial dysfunction and inducing irreversible tissue damage or cell death. Ischemia-reperfusion injury-triggered inflammatory cascade is initiated by the activation of CD1d-restricted invariant natural killer T (iNKT) cells, by the release of IFNγ and INFγ-inducible chemokines (CXCL9 and CXCL10), and by high numbers of lymphocytes expressing the chemokine receptor CXCR3 [202]. Thus, a vicious cycle exists between sickle RBC ROS production, vascular damage, and inflammation.

Both ROS released from sickle RBCs during hemolysis and released heme stimulate oxidative stress, which is associated with induction of the endothelial antioxidant enzyme heme oxygenase-1 (*HO-1*), an enzyme that stems from NF-κB pathway activation [203]. In SCD, overexpression of *HO-1* is involved in the deposition of pathological iron and mitochondrial damage [50,154]. Alternatively, oxidative stress-induced PECAM-1 phosphorylation [204] downregulates *HO-1* through the antioxidant transcription factor nuclear factor (erythroid-derived 2)-like 2 (Nrf2) [148,205]. Nrf2 plays a critical role in the cellular antioxidant response under oxidative stress conditions. Loss of Nrf2 function in Nrf2 knockout transgenic sickle mice impacts γ-globin gene expression and the control of both oxidative stress and inflammation, and is associated with the phenotypic severity of SCD [148,206]. Nrf2 activation in monocytes/granulocytes and endothelial cells cooperatively contributes to the improvement of SCD pathology [207]. Thus, overproduction of ROS by enzymatic (xanthine oxidase, NADPH oxidase, and uncoupled endothelial nitric oxide synthase (eNOS)) and non-enzymatic pathways (Fenton chemistry) promotes intravascular oxidant stress that can likewise disrupt nitric oxide (NO) homeostasis [208]. Cell-free hemoglobin also scavenges NO, which reduces NO availability in SCD [168], subsequently impairing endothelial-dependent vasodilation, thus altering blood cell adhesive function [209]; these are events leading to vascular complications. In addition, reduced NO bioavailability can be exacerbated by increased NO degradation caused by ROS, by decreased expression of eNOS, by the deficiency of substrates or cofactors for eNOS, and by an inappropriate activation of eNOS caused by impaired cellular signaling [210,211,212]. Kato et al. [213], in addition, reported positive associations between the level of plasma soluble adhesion molecules and the severity of pulmonary hypertension, which is a clinical manifestation of endothelial dysfunction in patients with SCD. Moreover, NETosis induced by extracellular heme-derived from lysed erythrocytes can cause endothelial activation and damage [177].

## 7. The Effect of ROS in Sickle RBCs on Hypercoagulation

SCD is characterized by a hypercoagulable state which was evidenced by an increase in markers of thrombin generation and platelet activation [121,158,214,215,216]. Although there is no direct evidence showing that oxidative stress causes hypercoagulability, ROS in sickle RBCs may well contribute to the development of SCD hypercoagulable state. Sickle cells are now recognized to play an essential part in promoting venous thrombosis [217,218,219]. RBCs activate the coagulation cascade and they contribute to thrombus formation via specific interactions [220,221,222] with plasma proteins, most notably fibrinogen [223], and with activated endothelial cells prior to platelet adhesion [218]. Increased RBC–endothelial cell interactions affect blood viscosity, leading to activation of the coagulation factors, consequently triggering vascular complications.

**Figure 3:**
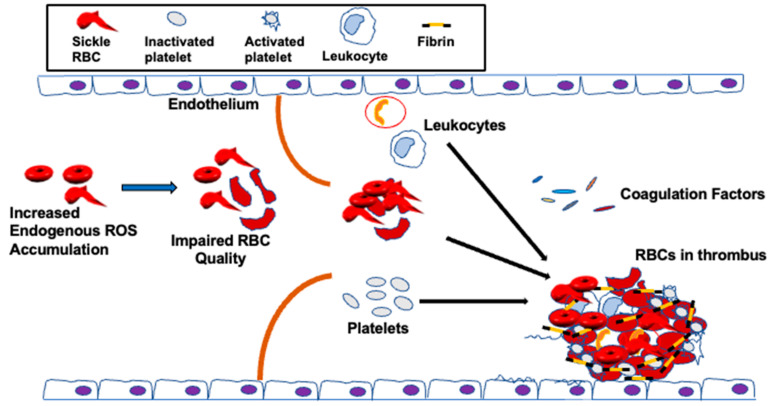
Potential contributions of sickle RBCs to venous thrombosis in SCD. Increased ROS levels in sickle RBCs impair membrane structure and function, leading to membrane integrity loss and reduced deformability, thus negatively modulating sickle RBC quality. This leads to a reduced flow of sickle RBC through the microcirculation. Venous thrombi form slowly in the presence of low flow or stasis and they are rich in both RBCs and fibrin. In the vasculature, sickle RBCs aggregate into stacked rouleaux structures, increasing blood viscosity. These sickle RBCs also adhere to the endothelium and participate in thrombin generation within thrombi. Once incorporated into the venous thrombus, sickle RBCs increase clot size and reduce both clot permeability and susceptibility to lysis. In SCD, sickle RBCs and sickle RBC-derived micro-particles also adhere to the vessel wall or extracellular matrix; activate endothelial cells, platelets, and other blood cells; and enhance local thrombin generation during thrombosis.

It has been postulated that RBC abnormalities, for instance, phospholipid asymmetry loss, can predispose SCD patients to thrombosis [156,157,224], despite the fact all other clotting factors are normal. Clinical and epidemiological studies have shown that loss of sickle RBC quality, which is negatively affected by excessive endogenous oxidative stress, is implicated in venous thrombosis [156,157,225]. The sickle RBC quality is also impaired by extracellular ROS [32,33,50,125,226] released by endothelial cells [227], neutrophils, and macrophages [228,229]. As a result, sickle RBCs are vulnerable towards oxidative stress-mediated damage due to the limited ability of the antioxidant system to neutralize accumulated ROS in access [30,230]. The un-neutralized ROS in the sickle RBC can, in turn, affect the structure of the membrane [9,65,231], thereby increasing blood viscosity and decreasing the RBC flow rate through the circulation [6,232,233], impairing RBC function in hemostasis and thrombosis [42,48] (Figure 3).

Importantly, PS exposure on RBCs also plays a crucial role in the development of vascular thrombosis [165,234,235,236,237]. Even a small fraction of RBC-exposing PS has been suggested to participate in thrombin generation. These RBC-exposing PS might explain 30–40% of the thrombin-generating potential of whole blood [238], uncovering the key implication of PS in thrombosis [239]. Correlations exist between PS levels on RBCs and the levels of prothrombin fragment F1.2, thrombin-antithrombin complex, plasmin-antiplasmin complex, and D-dimer in plasma, suggesting that increased PS exposure on RBCs may cause a procoagulant phenotype [239]. Our studies have implicated ROS accumulation within the sickle RBCs in inducing PS exposure on the cell surface [15]. Undeniably, lowering sickle RBC ROS production with Mn porphyrins, via likely suppressing RBC NOX activation [15], ameliorated eryptosis reflected by low PS exposed on sickle RBCs in sickle mice, indicating that high NOX-dependent ROS levels in sickle RBCs may up-regulate PS exposure on the surface of these cells [27]. Increased NADPH oxidase-dependent ROS production within the sickle RBCs and exposure of PS both mediate adhesion of these cells to the vascular endothelium in vitro and in vivo [15,27,79]. Elevated intracellular Ca^2+^ accumulation is another factor inducing PS externalization on RBCs via activation of scramblase, a molecule involved in the translocation of PS between the two monolayers of a lipid bilayer of the cell membrane [240,241]. Intracellular ROS stimulates several Ca^2+^ transporters localized in the plasma membrane, causing high levels of Ca^2+^ accumulation within the cell and vis versa [242,243]. Ca^2+^ in RBCs was believed to be limited to its contribution to RBC aging and clearance [244,245,246]. Yet, RBC Ca^2+^ is crucial in regulating various processes including O_2_ transport [247], rheology [248], and clotting by altering RBC rheological properties and RBC aggregation [249,250]. Thus, abnormal RBC Ca^2+^ homeostasis triggers not only venous thrombosis but severe life-threatening systemic pathologies as well.

Reduced blood flow due to inferior sickle RBC quality caused by increased endogenous ROS levels [251,252] is another important factor precipitating thrombotic processes [5,252,253]. Reduced blood flow or blood stasis permits RBCs to form aggregates [254], consequently triggering venous thrombosis [255]. Defective RBCs also adhere to the endothelium, interact with leukocytes, or aggregate with other cells to form multi-cellular aggregates. These adhesive and functional interactions play a significant role in thrombosis [15,113,140,218,256]. Additionally, reduction in local flow allows sickle RBCs to decrease the wall shear stress, which lowers NO release [6,257,258,259,260]. Deficiency in NO stimulates interactions of platelets with the vascular endothelium and/or injury-exposed sub-endothelial matrix [261,262,263]. Lowering bioavailability of NO by sickle RBCs through the release of hemoglobin also induces platelet aggregation [168,264]. As such, reduced NO bioavailability could be involved in the high platelet activation [265] observed in SCD patients, documented by increased expression of platelet activation markers, P-selectin, CD63, activated glycoprotein IIb/IIIa, plasma soluble factor-3 and factor-4, β-thromboglobulin, and platelet-derived soluble CD40 ligand [266,267]. Furthermore, RBCs control platelet hemostasis [268]. These enucleated cells support shear-induced platelet adhesion by mainly enhancing the transport of flowing platelets to bind to different surfaces [269]. Platelets then become activated, leading to platelet FasL exposure that activates FasR on RBCs responsible for externalization of PS on the RBC membrane, consequently hastening thrombin formation [270]. Activated platelets trigger thrombin generation by providing a major site for assembly of the tenase (FIXa and FVIIIa) and prothrombinase (FXa and FVa) complexes [271], and by directly participating in thrombus formation. Sickle RBCs binding via ICAM-4 to integrins present on leukocytes (CD11-CD18) and on platelets (alpha2beta4) offers, in addition, a surface, which can be involved in thrombosis [144]. RBC binding to platelets activates the α_IIb_β3 integrin receptor and the expression of P-selectin on platelets, which strengthen platelet aggregability and increase platelet recruitment [272]. Platelet aggregation and degranulation are triggered by ATP and ADP, released by RBCs in response to chemical and physical mechanisms [273]. In SCD, higher levels of β-thromboglobulin (β-TG) and platelet factor 4 (PF4) have been documented [274]. Platelet activation is associated with thrombosis and pulmonary hypertension in SCD patients [275].

**Figure 4:**
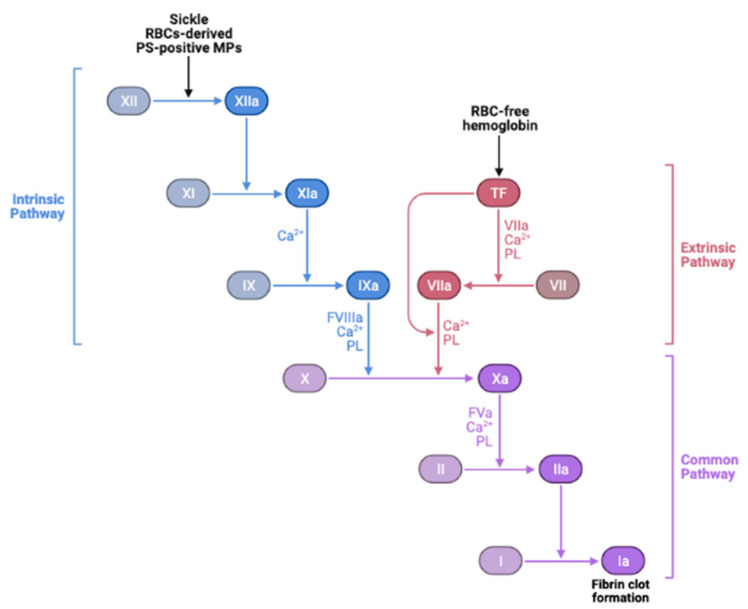
Activation of the coagulation cascade by sickle RBC-derived microparticles and tissue factors. Phosphatidylserine-positive microparticles (PS-positive MPs) derived from sickle RBCs and sickle RBC-free hemoglobin-induced tissue factor (TF) release enhances thrombosis in SCD by activating the coagulation cascade.

Furthermore, MPs or micro-vesicles (MVs), also called microscopic extracellular membranes structures, are implicated in various biological functions such as thrombosis and hemostasis [276,277], as well as inflammation [278]. Membrane micro-vesiculation is a physiologic process for mature RBCs and it is a well-regulated mechanism [279]. This process may contribute to irreversible membrane-carrying hemoglobin loss or to exocytosis of damaged cell components in RBCs [280]. Most RBC-derived MPs expose PS [156,280]. Excess ROS-induced PS exposure on sickle RBCs may accelerate the generation of MPs, subsequently promoting prothrombotic events in SCD. The abnormal hemoglobin S autoxidation is involved in membrane instability and MP shedding [281]. In this disease, MPs number has been found to correlate with the rate of intravascular hemolysis, implying that intravascular hemolysis may cause release into the bloodstream of sickle RBC-derived MPs as well [160,277] and with the degree of activation of coagulation [72,158]. PS-positive MPs potentiate thrombin generation [72,158,282] via FXI activation [158,277,283], while activated FXII is the main factor in the coagulation cascade, possibly involving the PS-mediated mechanism [284]. It is also suggested that RBC membranes can activate factor IX that may serve as a trigger of coagulation [285]. Thus, RBC-derived MPs could be considered as a potential therapeutic target to treat hemostatic disorders especially because of their wide procoagulant activity [284]. Moreover, SCD is often associated with elevated plasma levels of fibrinogen, coagulation factor XI, and von Willebrand Factor (vWF) [158,286,287,288,289]. Sickle RBCs can bind to fibrinogen via the RBC membrane, specifically β3 integrin, or CD47, or both, leading to RBC aggregation [131,223,289,290,291]. It is therefore likely that oxidative stress in sickle RBCs in cooperation with the “prothrombotic” hemostatic profile accelerate thrombotic events in SCD. Nonetheless, the induction of the antioxidant *HO-1* can offset the chain reaction created by oxidative stress-induced RBC-derived MPs, as well as the release of heme and iron, and may protect against tissue injury, consequently reducing the risk for recurrent venous thrombosis. In agreement with this notion, *HO-1* deficiency in mice prevents thrombus resolution, which amplifies the inflammatory reaction [292]. Other studies have shown that long GT-repeat alleles in *HO-1* gene (HMOX1) are associated with low *HO-1* anticoagulant activity and, hence, a high risk of thrombosis [293]. The instigated disruption and activation of the endothelial barrier by cell-free hemoglobin can also lead to exposure or release of prothrombotic proteins, such as collagen, tissue factor (TF), vWF [294,295], and chemotactic proteins such as cytokines and surface adhesion molecules [296], into the blood that support platelet aggregation, leukocyte recruitment, and thus further coagulation (Figure 4).

Formation of NETs provides an extra scaffold and stimulus for thrombus formation. NETs can cause platelet adhesion, activation and aggregation, erythrocyte recruitment, and fibrin deposition [297]. In addition, cell-free heme enhances and binds to TF on macrophages, promoting TF-dependent coagulation activation [298]. TheTF pathway inhibitor (TFPI), the only physiologic regulator of TF activity, can be inhibited by oxidative stress and can exert a procoagulant effect [299]. In SCD, extracellular ROS may further trigger a procoagulant state through oxidative modifications of proteins involved in coagulation. ROS directly inactivate the anticoagulant protein C [300] and its upstream agonist thrombomodulin [301]. ROS exert a prothrombotic role by oxidizing fibrinogen, accelerating the conversion to fibrin [302], and decreasing thrombin-binding to anticoagulants, the antithrombin III-heparin complex, and thrombomodulin [303]. ROS also reduce the heparin-binding capability of antithrombin [304], and can shed P-selectin. Circulating levels of P-selectin are associated with an increased risk of venous thromboembolism [305]. Additionally, ROS directly act as chemo-attractants for neutrophils [306]. Thus, RBC oxidative stress-impaired mechanical properties, deformability, and blood rheology, together with compromised RBC-exposing PS phagocytosis and hemolysis, can trigger venous prothrombotic events in SCD.

## 8. The Role of ROS in Sickle RBCs in the Activation of the Complement System

**Figure 5:**
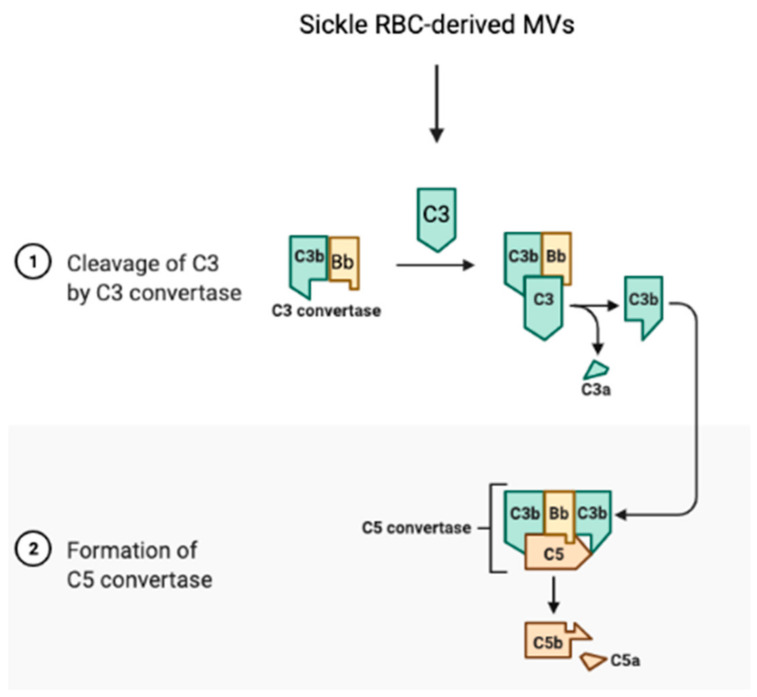
Activation of the complement system by sickle RBC-derived micro-vesicles in SCD. Micro-vesicles (MVs) derived from sickle RBCs can initiate activation of the complement system by cleaving C3 and C5 with C3 convertase and C5 convertase, respectively, leading to the release of the anaphylatoxins C3a and C5a, respectively.

The complement system links the coagulation cascade with the immune system, a cross-talk that is critical for the maintenance of homeostasis [307]. In SCD, the sickle RBC is a critical factor of activation for the complement system, which contributes to thrombosis. Heme-loaded sickle RBC-derived MVs activate the innate immune complement system and cause an inflammatory reaction, leading to the cleavage of complements C3 and C5, to the release of anaphylatoxins C3a and C5a, respectively, and to the formation of C5b-9, the terminal membrane attack complex (Figure 5) [161,308].

Activation of this cascade may stimulate procoagulant activity involving platelet prothrombinase [309,310]. The complex C5b-9 promotes the release of platelet factor V and prothrombinase complex assembly, hence potentiating the effects of thrombin on prothrombinase activation [309]. The anaphylatoxin C5a could enhance TF expression by endothelial cells and neutrophils [311,312], promoting extrinsic coagulation pathway activation. C5a, a potent proinflammatory mediator, activates platelets, endothelial cells, and leukocytes, all of which play a role in the vaso-occlusion in SCD [313]. Clinical studies have demonstrated significant elevated complement activation markers in sera of SCD patients and high sickle RBC-bound C3 levels [314], suggesting a possible role for abnormal RBCs in complement system activation, triggering thrombosis [315]. Further studies have implicated RBC-derived MVs, a relevant form of the heme carrier, and heme in activation of the alternative and terminal complement pathway, which were initiated on the endothelial surfaces to activate the thrombotic cascade [161,277,314]. Hemoglobin, though, triggers rapid P-selectin, C3a receptor (C3aR), and C5a receptor (C5aR) expression, as well as down-regulation of CD46 on endothelial cells, which are processes associated with inflammation and organ injury, eliciting subsequent blood coagulation [161]. P-selectin appears to drive complement attack on endothelial cells in a TLR-4/heme-dependent manner [316].

## 9. Targeting Oxidative Stress in Sickle Red Blood Cells

Numerous studies have attempted to reduce oxidative stress, thus enhancing antioxidant defenses. As reported above, in SCD, erythrocytes have an environment of continuous pro-oxidant generation due to both activation of NOX enzymes and hemoglobin autoxidation, which represent a major and quantitatively significant source of oxidative stress. Here, we report the most promising antioxidant therapeutic strategies that can directly affect sickle RBCs, showing a benefit in reducing oxidative stress parameters in SCD mouse models and in clinical trials in SCD patients.

*L-Glutamine*. L-Glutamine is an essential amino acid required for synthesis of the pyridines for nucleotides, including nicotinamide adenine dinucleotide (NAD) and glutathione, as well as glutamate. Oral administration of L-glutamine in SCD patients has been approved on July 2017 by the Food and Drug Administration (FDA) [317]. The NADH:[NAD⁺ + NADH] (redox) ratio in sickle RBCs is lower than in normal RBCs, consistent with oxidative stress; therefore, increasing glutamine availability is important as a therapy in SCD [318]. In addition, RBC total glutathione and glutamine levels were significantly lower in SCD patients than in healthy volunteers, and the ratio glutamine:glutamate correlated inversely to tricuspid regurgitant jet velocity, suggesting that a decrease in RBC glutathione and glutamine levels play a role in the pathogenesis of pulmonary hypertension in SCD [319]. In a sickle mouse model, glutamine levels were directly related to cerebral blood flow [320]. In SCD patients, oral L-glutamine was associated with increased NADH and reduced RBC adhesion to the endothelium [321]. The mechanism underlying this effect is still unclear; yet, the improvement of NAD redox potential may protect RBC from oxidant damage and from the consequent stimulation of inflammation and expression of adhesion molecules. In phase III randomized, double-blind, controlled trials, L-glutamine at 0.3 g/kg/dose twice daily was effective in reducing painful episodes in patients with SCD as well as hospitalizations, and this drug was well-tolerated [322,323]. However, L-glutamine was only tolerated in two-thirds of patients and failed to improve anemia and hemolysis [323].

*N-Acetylcysteine*. N-acetylcysteine (NAC) is converted to L-Cysteine, the precursor of GSH, which, as mentioned above, is reduced in RBCs of SCD patients [31]. Reduced RBC GSH appears to be due to an increase in the excretion of the oxidized GSSG form. NAC is an important antioxidant that influences inflammation and vasomotor function [324]. Studies have shown that the treatment of blood cells with NAC increases the intracellular concentration of the reduced form of GSH and decreases oxidative stress both in vitro and in vivo [325]. In a phase II double-blind, randomized clinical trial (NCT01800526), NAC inhibited dense cell formation, restored glutathione levels towards normal, and decreased vaso-occlusive episodes at a well-tolerated dose of 2,400 mg per day [326]. Furthermore, in an open-label randomized pilot study, NAC at both 1,200 and 2,400 mg doses seemed to decrease the cell membrane phosphatidylserine expression, marker of peroxidative damage to the erythrocyte inner membrane, and plasma levels of advanced glycation end-products (AGEs) as well as cell-free hemoglobin after 6 weeks of NAC treatment in both dose groups [33]. Thus, NAC may reduce SCD-related oxidative stress.

*α-Lipoic Acid and Acetyl-L-Carnitine*. α-Lipoic acid (LA) and acetyl-L-carnitine (ALCAR) have antioxidant properties. LA and ALCAR are in phase 2 trials in patients with SCD (NCT01054768). LA can induce GSH synthesis through inducing Nrf2-dependent transcription of γ-glutamyl cysteine ligase (GCL) [327]. GCL controls the rate of GSH synthesis [327]. The beneficial effect of ALCAR, however, might occur by facilitating the entry of long-chain fatty acids into mitochondria and decreasing lipid peroxidation in tissues [317]. ALCAR might be capable of maintaining the normal shape of RBCs and decreasing peroxidative damage [324,327]. Combination treatment with LA and ALCAR has been shown to have a synergic antioxidant effect in human fibroblast exposed to iron in excess [327].

*Manganese (Mn) porphyrins*. In the SCD mouse model, the Mn porphyrins, namely MnBuOE and MnE, can reduce vaso-occlusion by undoubtedly undergoing intricate interactions with numerous intracellular redox-sensitive pathways not only in sickle RBCs by suppressing NOXs, MEK1/2, ERK1/2, and GRK2-signaling [15], but also in leukocytes and the endothelium [27,328]. Studies have shown that Mn porphyrins act both intracellularly—the cytosol, nucleus, and mitochondria—and extracellularly, mimicking extracellular SOD, MnSOD and Cu, and ZnSOD [328,329,330,331]. MnE and MnBuOE have similar ability to interact with ROS [329]. In addition to their role as powerful SOD mimics, being catalysts of superoxide dismutation, Mn porphyrins can rapidly react with a number of other species and they have been suggested to be involved in thiol-signaling [329,330,331,332,333,334], catalytically oxidizing protein cysteines in a GPx fashion employing H_2_O_2_ and GSH [328,330,333]. Mn porphyrins also modify the activity of transcription factors, such as NF-кB, and, in turn, NOXs, both in normal tissue injuries and cancer [328,333,334,335,336]. In a similar manner, multiple MAPKs, including ERK1/2, AKT, c-Jun N-terminal kinase (JNK), and p38, seem to be oxidized by Mn porphyrins in tumor studies and are subsequently inactivated [328,337,338,339]. For instance, MnE can suppress NOX4 upregulation (presumably via the NF-кB pathway) due to a radiation-induced increase in ROS levels [340]. MnBuOE reportedly activates Nrf2 presumably via oxidizing cysteines of Keap1, consequently up-regulating endogenous antioxidative defenses [341]. Inhibition of these different mechanisms by Mn porphyrins would result in decreased ROS levels. Thus, the wealth of the efficacy and toxicity data validated the progress of several Mn porphyrins toward multiple clinical trials [330,342,343].

## 10. Conclusions

SCD is a multifactorial disease in which the sickle RBC is the root cause of the disease pathogenesis. Oxidative stress in sickle RBCs due to NOX enzyme activation, along with hemoglobin autoxidation, plays an essential role in SCD pathophysiology. The production of ROS in sickle cells promotes cell adhesion, vaso-occlusion, endothelial dysfunction, hemolysis, vascular inflammation, activation of the coagulation cascade and the complement system, and organ injury. These pathological changes lead to the production of more ROS, further exacerbating the disease. Using antioxidant reagents that specifically target oxidant production in sickle RBCs could reduce the detrimental effects of oxidative stress in SCD. The recent discoveries in developing novel drugs have led to improved survival and decreased morbidity in patients with SCD, but an improved understanding of oxidative stress and its regulation, especially in sickle RBCs, could lead to targeted therapies that should improve outcomes for this patient population. Mn porphyrins, for instance, which can reduce oxidative stress in sickle RBCs as well as in leukocytes and in the endothelium, have shown, in general, remarkable therapeutic effects in the SCD mouse model. The potential therapeutic effect of Mn porphyrins to prevent the development of organ complications deserves future investigation.
